# Unstable power threatens the powerful and challenges the powerless: evidence from cardiovascular markers of motivation

**DOI:** 10.3389/fpsyg.2015.00720

**Published:** 2015-05-27

**Authors:** Daan Scheepers, Charlotte Röell, Naomi Ellemers

**Affiliations:** ^1^Social and Organizational Psychology Unit, Institute of Psychology, Leiden UniversityLeiden, Netherlands; ^2^Leiden Institute for Brain and Cognition, Leiden UniversityLeiden, Netherlands

**Keywords:** social power, social interaction, cardiovascular responses, challenge, threat

## Abstract

Possessing social power has psychological and biological benefits. For example, during task interactions, people high in power are more likely to display a benign cardiovascular (CV) response pattern indicative of “challenge” whereas people low in power are more likely to display a maladaptive CV pattern indicative of “threat” (Scheepers et al., 2012). Challenge is marked by high cardiac output (CO) and low total peripheral resistance (TPR), while threat is marked by low CO and high TPR (Blascovich and Mendes, 2010). In the current work we addressed a possible moderator of the power-threat/challenge relationship, namely the *stability* of power. We examined the influence of the stability of power (roles could or could not change) on CV responses during a dyadic task where one person was the “chief designer” (high power) and one person was the “assistant” (low power). During the task, different CV-measures were taken [CO, TPR, heart rate, pre-ejection period). Whereas participants in the unstable low power condition showed a stronger tendency toward challenge, participants in the unstable high power condition showed a stronger tendency toward threat. Moreover, participants in the stable low power condition showed CV signs of task *dis*engagement. Results are discussed in terms of the importance of contextual variables in shaping the relationship between power and benign/maladaptive physiological responses.

## Introduction

Social power—the ability to allocate or withhold resources from others—is one of the primary factors determining behavior in interpersonal, intra-group and inter-group settings (Cartwright, 1959; Mulder, 1977; Ng, 1980; De Waal, 1982; Galinsky et al., 2003; Keltner et al., 2003; Fiske and Berdahl, 2007; Guinote, 2007). Power not only defines the structural relationship between people (who “leads” and who “follows”), it also determines the affective and physiological responses of power holders and their subordinates, the cognitive strategies they use for solving problems, and the (non-verbal) behavior they direct to each other (Anderson and Berdahl, 2002; Keltner et al., 2003; Tiedens and Fragale, 2003; Anderson and Galinsky, 2006; Smith and Trope, 2006; Carney et al., 2010).

On the basis of the limited control they have, it seems only logical to assume that the powerless experience more stress than the powerful. There is indeed evidence for this assumption, also at the psychophysiological level. For example, compared to those high in power, those low in power do often have higher levels of cortisol (Wirth et al., 2006; Carney et al., 2010; Mehta and Josephs, 2010) and tend to display a cardiovascular (CV) response profile indicative of threat (Scheepers et al., 2012; Akinola and Mendes, 2013; Kraus and Mendes, 2014). Over time, frequent occurrence of these physiological response patterns can have serious negative health consequences (Chen et al., 2010).

However, as we will elaborate in more detail below, it is not self-evident that people with high power are always better-off in terms of stress. In the current research we examine the influence of the *stability* of power on CV-indices indicative of threat and challenge during a dyadic task situation that is characterized by a power difference. Our central hypothesis is that when power is unstable the powerless are relatively more challenged, and the powerful are relatively more threatened.

In the past 15 years, research on power has flourished within the field of social cognition (Galinsky et al., 2003; Keltner et al., 2003; Fiske and Berdahl, 2007; Guinote, 2007). A leading hypothesis in this field has been that possessing high power leads to activation of the behavioral activation system (BAS), while possessing low power leads to activation of the behavioral inhibition system (BIS; Keltner et al., 2003; Smith and Bargh, 2008). Related models have linked power to goal activation and goal pursuit (Guinote, 2007) and to a more general inclination to take action (Galinsky et al., 2003). In research revealing evidence for these predictions, power has typically been manipulated by means of priming tasks or specific role assignments in a (dyadic) task situation. Based on these models and procedures, low power has for example been related to a closed body posture, negative affect, a focus on possible losses, concrete thinking, and the avoidance of risks, while high power has been related to a more open body posture, positive affect, a focus on possible gains, abstract thinking, and risk-seeking behavior (Anderson and Berdahl, 2002; Keltner et al., 2003; Anderson and Galinsky, 2006; Smith and Trope, 2006; Carney et al., 2010).

The basic hypotheses regarding power, approach, avoidance, and related motivational tendencies have also been examined at the psychophysiological level. For example, using EEG measurements, Boksem et al. (2012), found that research participants who were primed with high (vs. low) power displayed a relative increase in left-sided frontal brain activity, which has been related to activation of the BAS. Furthermore, research on the neuroendocrine correlates of power has shown that high power is related to low levels of cortisol and high levels of testosterone (Schultheiss et al., 2005; Wirth et al., 2006; Carney et al., 2010; Akinola and Mendes, 2013) which represents a marker of approach tendencies during demanding situations (Mehta and Josephs, 2010).

Previous work on CV responses to power differences is most relevant to the current research. For example, Van Kleef et al. (2008) demonstrated that high power led to stronger parasympathetic regulation of heart rate (HR; as measured by *respiratory sinus arrhythmia*; RSA) during a potentially stressful event. An increase in RSA is indicative of more effective down-regulation of negative affect in demanding situations. This finding is in keeping with research that has applied the biopsychosocial model of challenge and threat (BPS-CT; Blascovich and Mendes, 2010) to the psychology of power. The BPS-CT, which is outlined in more detail below, identifies specific CV indices of “challenge” and “threat” motivational states. Building on this model, Scheepers et al. (2012) showed that research participants primed with high power displayed a challenge response while participants primed with low power displayed a threat response. Akinola and Mendes (2013) obtained similar results during a dyadic cooperative task where one participant was the “leader” and one person was the “support person.”

The evidence summarized above may lead to the impression that people in relatively high power positions are in a quite comfortable position, while people low in power are more or less chronically threatened. This seems to be an oversimplification, however. Results from previous research suggest that there are important moderators for the relation between power and approach/avoidance tendencies, such as the perceived legitimacy and/or stability of the power difference (Magee et al., 2005; Maner et al., 2007; Lammers et al., 2008). For example, Lammers et al. (2008) showed that high power is only positively related to approach when the power had been obtained in a legitimate way. By contrast, when power differentials were based on an illegitimate procedure, low power was positively related to approach. This latter response has been interpreted as a sign of revolt by the powerless person. Regarding the stability of power, Maner et al. (2007) found that the classic effect that power leads to more risky decision-making was only observed when power relations were stable. Indeed, when power differentials were unstable the powerful people became more conservative in their decision-making.

The current research builds on this previous work but extends existing insights by addressing the more basic motivational and psychophysiological responses stemming from power (in)stability. Our central hypothesis is that the beneficial effects of high power (i.e., challenge) should only emerge when power relations are *stable*. When power differences are *unstable*—this should elicit threat in the powerful, as their privileged position is subject to change in the future, and challenge in the powerless, for whom there is scope to improve their position (see also Sapolsky, 2005).

Animal research provides indirect evidence for this hypothesis. In his seminal work on stress in primates, Sapolsky (2005) examined the relation between social rank and neuroendocrine response patterns. This research clearly indicated that rank in itself was a suboptimal predictor of maladaptive neuroendocrine stress profiles. Instead, what clearly predicted these stress profiles was the interaction between rank and the stability of ranks. When ranks were stable the low-ranked primates showed most physiological signs of stress, as might be expected. However, when ranks were unstable the highly ranked primates were the ones that showed most evidence of stress. In the current research we extend this prior work, by addressing the role of stability of interpersonal power relations in humans working together on a cooperative task.

Further evidence in support of the validity of our reasoning can be found in prior research on CV responses to the stability of status differences between different social groups (Scheepers and Ellemers, 2005; Scheepers, 2009; see Scheepers, 2013 for an overview). In both lab-created minimal group contexts, as well as in more naturalistic groups (where status differences were based on gender) members of low status groups were found to display more CV signs of threat when status differences between groups were perceived to be stable. By contrast, members of high status groups revealed more CV signs of threat when inter-group status differences were perceived to be unstable. This prior work is about differences in social status (instead of high vs. low control due to power differences) and addresses relations between groups instead of individuals. Nevertheless, it seems relevant for the current investigation because it examines how the stability of social relations impacts on threat. Moreover, this prior work has used a methodology that makes it relevant to the present investigation, namely, the assessment of CV indices of threat following the BPS-CT. This model is explained in greater detail in the next paragraph.

The BPS-CT identifies specific CV-markers of the motivational states of challenge and threat during so-called motivated performance situations (e.g., athletic performance, doing a math test, giving a speech). According to the BPS-CT, threat and challenge result from the evaluation of a motivated performance situation in terms of its demands (e.g., effort, uncertainty, danger), as well as the person’s resources (e.g., skills, knowledge, support, dispositions) to deal with these demands. When demands outweigh resources, a threat motivational state arises, whereas when resources approach or exceed demands, this induces a motivational state of challenge (Blascovich and Tomaka, 1996; Blascovich, 2008a,b; Blascovich and Mendes, 2010; Seery, 2013).

At the CV-level, challenge is marked by high cardiac output (CO, the amount of blood pumped out by the heart per minute), coupled with low total peripheral resistance (TPR, a measure of vascular resistance to blood flow), which enables the efficient mobilization and transportation of energy during motivated performance. Threat, by contrast, is marked by relatively high TPR and low CO, which leads to a less efficient mobilization and transportation of energy during motivated performance.

Two analytical strategies have been documented to interpret CV-data in the context of the BPS-CT. The first strategy is to examine *relative* differences in levels of CO and TPR *between* experimental conditions using (M)ANOVA. Relatively low levels of CO and high levels of TPR signal higher threat (and lower challenge) and vice versa. The second strategy is to examine *absolute* patterns of CV-reactivity *within* conditions, by examining increases and decreases in CV-responses compared to baseline levels. A pattern of threat CV-reactivity is indicated by significant increases in TPR in combination with unchanged CO, while a pattern of challenge CV-reactivity is indicated by significantly decreased TPR and increased CO (Blascovich and Mendes, 2010).

As indicated above, motivated performance situations constitute the context of the BPS-CT. A certain level of task engagement is required to be able to define a situation as involving motivated performance. As a check on whether task engagement is present, two additional CV-indices are commonly examined in research using the BPS-CT: HR and *pre-ejection period* (PEP). Whereas HR refers to the *pace* with which the heart pumps, PEP, representing a measure of left-ventricular contractility, is a measure of the *force* with which the heart pumps. PEP is the most direct measure of sympathetic nervous system influence on heart activity (Brownley et al., 2000) and has additionally been described as the most direct measure of task engagement (as defined as effort; Kelsey, 2012), and the superior CV measure to index BAS activation (Brenner et al., 2005). Within the BPS-CT, engagement is indexed by significant increases in HR and decreases in PEP, compared to baseline levels.

The BPS-CT has been validated in dozens of studies, and has provided a new motivational perspective on a variety of topics, ranging from social facilitation to inter-ethnic interactions (see Blascovich and Mendes, 2010; Seery, 2013 for overviews). In the current research we examine the influence of power stability on CV threat – challenge responses, in line with the analysis provided by the BPS-CT model.

In the current study participants engaged in a cooperative, dyadic, computer-mediated task: designing and furnishing a house using a computer simulation program (“Sweet home 3D^®^”). During the task, one person would be assigned the “chief designer” role (high power), and one person the “assistant” (low power). The stability of the power role was manipulated by specifying that the roles would remain the same for the duration of the task, or that the roles could possibly change after a first phase of the task. We predicted that when power was said to be stable the person with the low power role would be more threatened, and the person with the high power role would be more challenged; when power was said to be unstable the person with the high power role would be more threatened, and the person with the low power role would be more challenged.

In addition to CV-measures, we also included several self-report measures to capture some relevant outcome measures in the psychology of power. Specifically, we assessed positive and negative affect, regulatory focus (promotion and prevention focus; Higgins, 1997), action tendencies, and optimism (Anderson and Berdahl, 2002; Galinsky et al., 2003; Anderson and Galinsky, 2006).

## Materials and Methods

### Participants and Design

Participants were 80 students (69% women; age: *M* = 21 years, range = 18–29) at Leiden University. They received €4 or course credits for their participation. Participants were randomly assigned to one of the four conditions of the 2(Power: Low vs. High) × 2(Stability: Stable vs. Unstable) design.

During the debriefing two participants displayed suspicion about the manipulations and their data were excluded from further analysis. In addition, the data of the first six participants were not included in the analyses due to a programming error in the manipulation check procedure; these participants were presented with incorrect feedback on their responses to the manipulation check items. Finally, due to signal loss or motion artifact we had missing or incomplete blood pressure data for 15 participants, and missing, incomplete or unscorable impedance-cardiographic (ICG) and/or electrocardiographic (ECG) data for three participants. The participants that were included in the analyses were still evenly divided across conditions as evident from non-significant χ^2^-tests on the number of cases included in the analyses on the different dependent variables, χ^2^s < 1.12, *p*s > 0.773.

### Cardiovascular Recording

Throughout the experimental session we continuously measured, ECG and blood pressure signals using a Biopac MP150 system (Biopac Systems Inc., Goleta, CA, USA). Physiological data was stored using *Acqknowledge* software (Biopac Systems, Goleta, CA, USA) and the ICG was scored using AMS-IMP software (Vrije Universiteit, Amsterdam, Netherlands).

For measuring ICG, the Biopac NICO100c module was used, together with four spot electrodes. Two electrodes were placed at the back of the neck (one at the base of the neck, the other ∼5 cm higher), and two spot electrodes were placed at the lower back (again ∼5 cm separated from each other). The distance between the two inner electrodes was ∼30 cm. The two outer electrodes injected a small (400 μA) alternating current while the two inner electrodes measured the voltage developed through the thorax volume. As output the NICO100c provided measures of basal impedance (Z_0_) and the rate of change in impedance (dZ/dt) which, in combination with the ECG, can be used to derive measures of PEP and CO. For determining CO we first calculated Stroke Volume (SV: the amount of blood pumped out on a single heartbeat), making use of the Kubicek formula (see Sherwood et al., 1990); Z_0_ was entered in the formula as a constant, for which we took the mean Z_0_ of both baseline and speech task. In turn, CO was calculated by multiplying HR and SV.

Electrocardiography was measured using an ECG100 module and two electrodes: one placed at the suprasternal notch above the top of the sternum, and one at the apex of the heart, on the left lateral margin of the chest approximately at the level of the processus xiphodius. We did not use a ground electrode as the participant was already grounded via the NICO100c. The ECG was used to determine HR and, in combination with the ICG, PEP.

Blood pressure was measured continually using a Vasotrac^®^ APM205a blood pressure monitor. This apparatus is equipped with a wrist sensor, which was placed over the radial artery of the participant’s non-preferred hand to measure the pulse wave from the radial pulse. Every 15 s a measurement was taken. The monitor provided a measure of mean arterial pressure (MAP) which, in combination with CO, was used to calculate TPR, using the following formula: TPR = (MAP/CO) × 80.

In addition to examining CO and TPR separately, we also calculated a combined threat-challenge index (TCI) by calculating *Z*-scores of CO and TPR, then multiplying TPR with -1 and summing the result with the CO *Z*-score (Blascovich et al., 2004; Kassam et al., 2009; Seery et al., 2010). Higher scores on the resulting index—which maximizes the reliability of the CV measures (Seery et al., 2010)—indicate a greater challenge motivational state, whereas lower scores indicate a greater threat motivational state.

### Procedure and Independent Variables

The research was conducted in conformity with the guidelines of the Ethics Committee of the Department of Psychology of Leiden University. The whole experiment was run on computers such that all information, tasks and manipulations were delivered via the computer. After arriving at the lab, the participant was seated in a cubicle, where sensors for physiological recording were applied. As part of the cover story relating to the power manipulation (see below) participants first completed a “leadership questionnaire” which consisted of a mixture of the eight items of the sense of power scale (Anderson and Galinsky, 2006; example: “If I want to, I get to make the decisions”) and nine items from the multi-factor leadership questionnaire (Bass and Avolio, 1990; example: “I can inspire others”). After completing the items, 5 min of baseline CV-responses were collected during which the participant sat quietly and relaxed.

After the baseline period the participants were told that the study was about performance on a dyadic computer-mediated task. Although we led participants to believe that they would collaborate with another participant who was in an adjacent cubicle, in fact this person did not exist, and the experiment ended before participants actually performed on the task. The task consisted of first designing and then partly furnishing a house on the basis of the “Sweet home 3D^®^” application. Participants were told that they would work together on the task, but that one person would take the role of “chief designer” (high power) and that the other person would take the role of “assistant” (low power). Before explaining the roles in more detail, participants were first provided with the opportunity to explore the “Sweet home 3D^®^” application for some moments. The application was started, to allow participants to explore the different options and practice with the interface.

Participants were then assigned a role during the task, apparently on the basis of their scores on the leadership questionnaire (see Galinsky et al., 2003, Experiment 1 for a similar manipulation). In fact, the role assignment was made randomly. In the high power condition participants were told that they, as “chief designer,” could design the house according to their wishes, and would instruct the assistant who would in turn carry-out the practical steps within the “Sweet home 3D^®^” program. In addition, the chief designer would determine the dimensions that should be used to evaluate the product, and would evaluate the assistant’s performance. Participants in the low power condition (“assistants”) were told that they would receive instructions from the chief designer and that they had to carry-out these orders to design the house according to the designer’s wishes. Furthermore, participants in the low power condition were told that the chief designer would decide how the task would be evaluated, and would also evaluate the assistant’s performance.

After manipulating power differences in this way, the stability of the power roles was manipulated. It was explained that the task would consist of two rounds: first designing the house, and then furnishing the house. In the stable condition participants were told that the roles (chief designer, assistant) would remain the same for the duration of both rounds of the task. In the unstable condition, participants were told that the power roles could possibly change after the first round, depending on “how the process of designing and building the house goes” (see Maner et al., 2007; Sligte et al., 2011 for similar manipulations of power stability).

After the manipulations of power and stability were induced in this way, participants delivered a short speech in front of the webcam. This represented the motivated performance situation we focused on with regard to CV-indices of challenge and threat (see Mendes et al., 2002; Weisbuch-Remington et al., 2005; Scheepers et al., 2012 for similar tasks). In the high power condition, the chief designer had to provide orders to the assistant in the speech, on how the house should be built. These instructions were said to be send to the assistant. In the low power condition the assistant could provide his or her view on the task, and indicate how they would like to see the house built in case they would have been in charge. This video was said to be recorded for “control purposes” – it would not be submitted to the chief designer. After the speech, participants completed the self-report measures (see below) and then learned that the session had ended, after which they received a debriefing via the computer. The debriefing was concluded with the information that participants should open the door of the cubicle and call the experimenter. After the experimenter had removed the electrodes, the participant was verbally probed for suspicion and was given the opportunity to ask further questions. Finally, participants were compensated for their participation, and then dismissed.

### Measures

Just before delivering the speech participants completed several items that were included to check the manipulations. First, participants were asked to indicate their role during the task by clicking on one of two buttons which were labeled: “chief designer” and “assistant,” respectively. In addition, participants completed three items measuring how much power they had in designing the house (e.g., “How much control do you have in designing the house?” α = 0.94). Responses to these latter questions were given on seven-point scales with 1 (“very little”), and 7 (“very much”) as end points.

The perceived stability of the power roles was checked by asking participants to indicate whether they could possible gain the chief designer role (in the low power condition) or whether they could possibly lose the chief designer role (in the high power condition). Participants responded by clicking on one of two buttons, which were labeled: “yes, I can gain [lose] the position of chief designer” and “No, I cannot gain [lose] the position of chief designer,” respectively.

The primary dependent measure was the CV-reactivity during the speech. However, just after the speech we also administered several self-report measures. Responses to the items were recorded on seven-point scales with 1 (“very little”), and 7 (“very much”) as end points. Positive and negative affect was measured by asking participants to indicate to what extent they experienced the following feelings and emotions: Happy, active, determined, positively challenged, alert, inspired, and strong (*positive affect*, α = 0.71), and threatened, scared, irritated, upset, and dejected (*negative affect*, α = 0.85). *Promotion focus* was measured using two questions (e.g., “I see the goal of building the house as well as possible primarily as an ideal,” *r* = 0.29, *p* = 0.009). *Prevention focus* was also measured using two questions (“I see the goal of building the house as well as possible primarily as an obligation,” *r* = 0.42, *p* < 0.001). *Optimism* was measured using three questions (“I feel certain about the success of this project”; α = 0.88). *Action-readiness* was measured using five items (e.g., “I’m prepared to take action”; α = 0.80). Finally, for control purposes we also assessed the expected *cooperation* during the task using two items (e.g., “I think that the cooperation will go smoothly,” *r* = 0.60, *p* < 0.001).

## Results

All data were analyzed using 2(Power: Low vs. High) × 2(Stability: Stable vs. Unstable) ANOVAs and ANCOVAs, except where indicated otherwise. Different number of degrees of freedom across tests are due to that for different variables we had different numbers of missing or excluded cases^1^.

### Data Screening and Checks

On the dichotomous checks, only one participant indicated his/her role in the team incorrectly (power check) and only one participant indicated the stability of the roles incorrectly (stability check). The participants who gave incorrect responses were prompted with the correct response before proceeding with the experiment; therefore, the data of all participants were retained the in main analyses reported below.

Analyses of the power manipulation check scale only revealed a significant main effect for power, *F*(1,68) = 241.48, *p* < 0.001; $\eta_{p}^{2}$ = 0.780 (other *F*s < 2.13, *p*s > 0.149). Participants in the high power conditions experienced more power (*M* = 6.23, SD = 0.90) than participants in the low power conditions (*M* = 2.09, SD = 1.33). We conclude that the manipulations have been successful.

### Cardiovascular Responses

In line with standard practice, mean levels of HR, PEP, CO, TPR, and TCI were calculated for the last minute of the baseline period and the first minute of the speech. The resulting scores were then examined for outliers, which were defined as values 3.3 SD greater or smaller than the mean. There was one outlier on baseline CO and there were two outliers on baseline TPR; these cases were assigned a value of 1% higher than the adjacent non-extreme value (see Weisbuch-Remington et al., 2005; Van Beest and Scheepers, 2013 for a similar procedure).

#### Task Engagement

The means and standard deviations for HR and PEP during baseline and speech task period are presented in **Table 1**. Overall there were significant increases in HR from baseline to speech task (*M*_baseline_ = 76.64; *M*_speech_ = 86.49), *t*(68) = -8.89, *p* < 0.001, and decreases in PEP from baseline to speech task (*M*_baseline_ = 122.32; *M*_speech_ = 111.01), *t*(68) = 5.86, *p* < 0.001, indicating task engagement and thus the requirements are met for a further interpretation of CV-reactivity in terms of challenge and threat.

**Table 1:** Heart rate (HR) and pre-ejection period (PEP) during baseline and speech task as a function of power and stability.

	Low Power		High Power	
	Unstable	Stable	Unstable	Stable
**Heart rate**
*M* (SD) Baseline *M* (SD) Speech *M* (SD) Reactivity 95% CI *t* *d*	77.61 (13.39) 87.04 (13.75) 9.43 (8.71) (4.96; 13.91) 4.47^∗∗∗^ 2.24	80.93 (13.44) 86.70 (11.45) 5.77 (10.68) (0.46; 11.08) 2.29^∗^ 1.11	75.04 (7.11) 87.22 (12.76) 12.49 (9.35) (7.68; 17.30) 5.51^∗∗∗^ 2.76	73.45 (15.09) 84.68 (10.20) 11.93 (6.60) (8.54; 15.32) 7.45^∗∗∗^ 3.73
**Pre-ejection period**
*M* (SD) Baseline *M* (SD) Speech *M* (SD) Reactivity 95% CI *t* *d*	121.65 (15.04) 104.00 (25.73) -17.65 (19.08) (-27.46; -7.84) -3.81^∗^ 1.90	126.67 (17.94) 122.67 (18.20) -4.00 (15.52) (-11.72; 3.72) -1.09 0.53	118.82 (18.31) 107.11 (17.22) -12.47 (14.96) (-20.27; -4.78) -3.44^∗∗^ 1.71	121.26 (15.89) 110.35 (15.24) -11.52 (2.29) (-17.69; -5.36) -3.97^∗∗^ 1.99

*The reported *t*-tests test CV-reactivity against 0 (i.e., baseline); ^∗^*p* < 0.05; ^∗∗^*p* < 0.01; ^∗∗∗^*p* < 0.001.*

A closer inspection of task engagement in the different conditions indicated that in both high power conditions participants showed clear signs of engagement (increased HR, decreased PEP). However, while clear signs of engagement were also present in the unstable low power condition, only moderate task engagement was observed in the stable low power condition. That is, although HR increased in the stable low power condition, PEP, which represents the more direct index for engagement/BAS (Brenner et al., 2005; Kelsey, 2012) did not differ significantly from zero in the stable low power condition.

**FIGURE 1:**
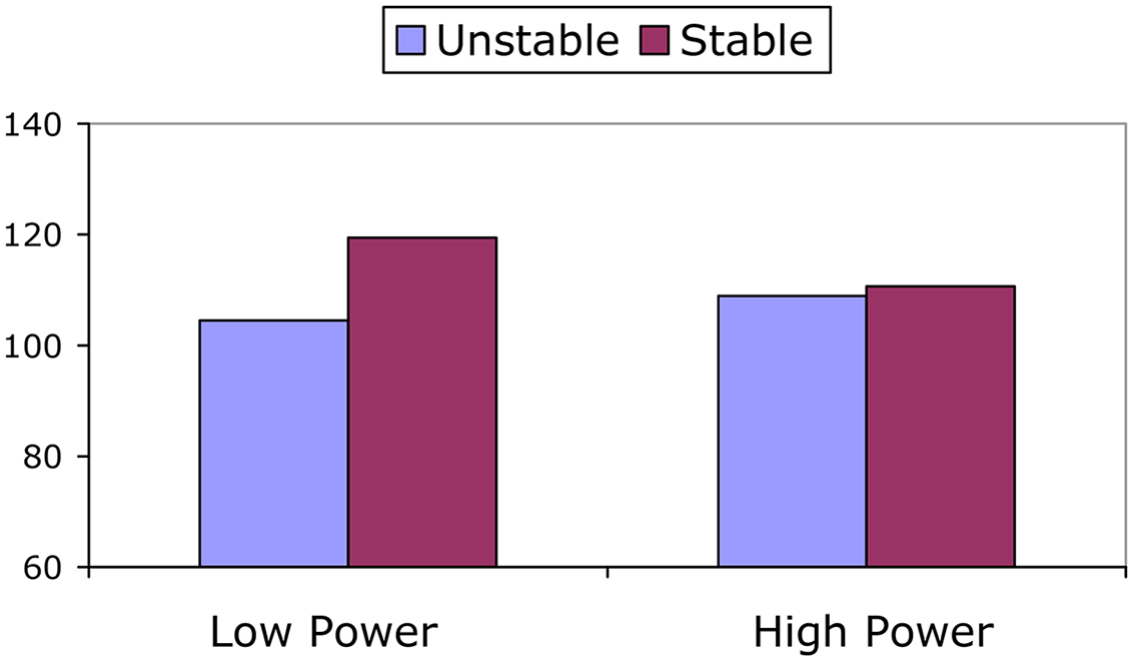
**Pre-ejection period (PEP) during the speech as a function of power and power stability.** Means are predicted means, controlled for baseline PEP.

To examine between-condition differences in HR and PEP we conducted separate ANCOVAs on HR and PEP during the speech task, with baseline HR and PEP as covariate in the respective analysis. In the case of HR this did not result in significant effects of power, stability, and their interaction, *F*s < 2.36, *p*s > 0.129. In the case of PEP this resulted in a significant main effect of stability, *F*(1,64) = 6.18, *p* = 0.026; $\eta_{p}^{2}$= 0.075, which was qualified by a marginally significant interaction between power and stability, *F*(1,64) = 3.31, *p* = 0.074; $\eta_{p}^{2}$ = 0.049. **Figure 1** displays the predicted means of PEP during the speech (controlling for baseline PEP), as a function of power and stability. Recall that lower PEP indicates more engagement. In keeping with the results of the within-condition analyses reported above, a test of the simple main effects showed that there was no difference in PEP between the stable and unstable high power condition, *F*(1,64) = 0.11, *p* = 0.739; $\eta_{p}^{2}$ = 0.002. However, participants in the stable low power condition had significantly higher PEP than participants in the unstable low power condition, *F*(1,64) = 8.49, *p* = 0.005; $\eta_{p}^{2}$ = 0.117. Moreover, when power was stable, PEP was somewhat higher in the low power condition than in the high power condition, *F*(1,64) = 2.92, *p* = 0.092; $\eta_{p}^{2}$ = 0.044. When power was unstable, there were no differences between the low and high power condition, *F*(1,64) = 0.74, *p* = 0.394; $\eta_{p}^{2}$ = 0.011. Together this indicates that participants in the low power condition *dis*engaged from the task when the position was stable. It is also noteworthy that–of all experimental conditions–participants in the unstable low power condition showed the strongest signs of engagement.

#### Challenge and Threat

Mean levels of CO and TPR as a function of power and stability are presented in **Table 2**. To examine *relative* differences in CV markers of challenge and threat, ANCOVAs on CO, TPR, and TCI during the speech were performed; baseline levels of the respective measure were added as covariates in the models. Regarding CO this analysis yielded a marginally significant main effect of stability, *F*(1,64) = 3.11, *p* = 0.083; $\eta_{p}^{2}$ = 0.046, which was qualified by a marginally significant interaction between power and stability, *F*(1,64) = 3.31, *p* = 0.073; $\eta_{p}^{2}$ = 0.049. A test of the simple main effects showed that there was no difference in CO between the stable and unstable high power condition, *F*(1,64) = 0.01, *p* = 0.983; $\eta_{p}^{2}$ < 0.001. However, participants in the unstable low power condition had significantly higher CO than participants in the stable low power condition, *F*(1,64) = 6.52, *p* = 0.013; $\eta_{p}^{2}$ = 0.092. There were no significant differences in CO between the low and high power condition when power was either stable or unstable, *F*s < 2.69, *p*s > 0.106.

**Table 2:** Cardiac output (CO) and total peripheral resistance (TPR) during baseline and speech task as a function of power and stability.

	Low Power		High Power	
	Unstable	Stable	Unstable	Stable
**Cardiac output**
*M* (SD) Baseline *M* (SD) Speech *M* (SD) Reactivity 95% CI *t* *d*	2.92 (0.82) 3.49 (1.26) 0.57 (0.72) (0.19; 0.94) 3.26^∗∗^ 1.63	2.65 (0.91) 2.76 (1.02) 0.11 (0.36) (-0.07; 0.29) 1.33 0.65	2.90 (0.81) 3.10 (1.00) 0.29 (0.48) (0.04; 0.54) 2.46^∗^ 1.23	2.79 (1.21) 2.86 (0.97) 0.27 (0.35) (0.09; 0.44) 3.18^∗∗^ 1.59
**Total peripheral resistance**
*M* (*SD*) Baseline *M* (*SD*) Speech *M* (*SD*) Reactivity 95% CI *t* *d*	2425 (849) 2509 (1090) 51 (422) (-193; 294) 0.45 0.23	3207 (1707) 3549 (2172) 328 (611) (-25; 680) 2.01 1.12	2361 (723) 2789 (1147) 282 (446) (13; 552) 2.28^∗^ 1.32	3066 (1728) 3211 (1597) -2 (182) (-107; 103) -0.05 0.03

*The reported *t*-tests test CV-reactivity against 0 (i.e., baseline); ^∗^*p* < 0.05; ^∗∗^*p* < 0.01; ^∗∗∗^*p* < 0.001.*

The only significant effect in the analysis on TPR was an interaction among power and stability, *F*(1,50) = 5.49, *p* = 0.023; $\eta_{p}^{2}$ = 0.099. When power was high, TPR was higher in the unstable than in the stable condition, *F*(1,50) = 4.07, *p* = 0.049; $\eta_{p}^{2}$ = 0.075. There were no differences between the unstable and stable low power condition, *F*(1,50) = 1.47, *p* = 0.231; $\eta_{p}^{2}$ = 0.029. Moreover, when power was stable, TPR was somewhat higher in the low power condition than in the high power condition, *F*(1,50) = 3.66, *p* = 0.061; $\eta_{p}^{2}$ = 0.068. When power was unstable there were no differences between the low and the high power condition, *F*(1,50) = 1.97, *p* = 0.167; $\eta_{p}^{2}$ = 0.038.

**FIGURE 2:**
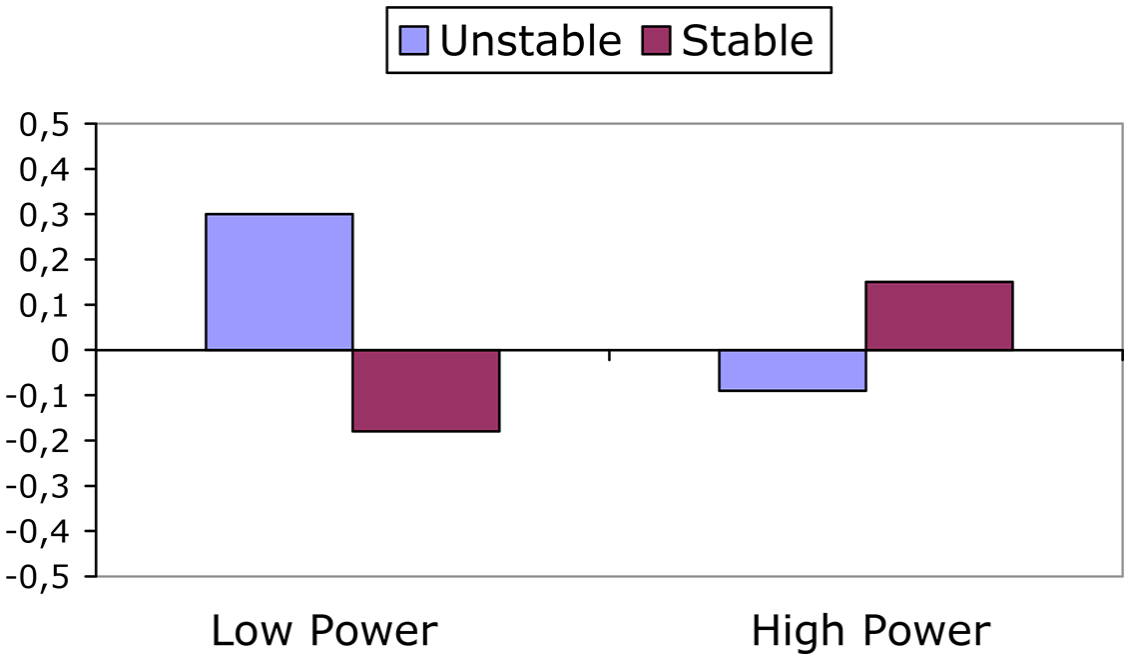
**Threat challenge index (TCI) during the speech as a function of power and power stability.** Means are predicted means, controlled for baseline TCI.

The only significant effect on the TCI was an interaction between power and stability, *F*(1,50) = 4.12, *p* = 0.048; $\eta_{p}^{2}$ = 0.076. This interaction is displayed in **Figure 2**. In the low power condition there was a stronger tendency toward challenge when power was unstable than when it was stable, *F*(1,50) = 3.61, *p* = 0.063; $\eta_{p}^{2}$ = 0.067. There were no other (marginally) significant simple main effects, *F*s < 2.41, *p*s > 0.127.

An examination of the *absolute* patterns of CV reactivity (see **Table 2**) provides some additional evidence for our main predictions. As can be seen in the table, there is only one condition where TPR significantly increases (in keeping with the threat pattern): the unstable high power condition. Despite that CO did also increase in this condition, the increased TPR corroborates with the finding that TPR was higher in the unstable high power condition than in the stable high power condition, and thus provides additional evidence for threat in the former condition. It is also noteworthy that despite significant increases in CO in the unstable low, and stable high power condition, there were no significant decreases in TPR. Thus, although there is evidence for relatively more challenge in these two conditions (see **Figure 2**), in absolute terms we cannot speak of a “full blown” challenge response. We will return to this issue in the discussion.

### Self-Report Measures

The mean and SD for the different self-report measures (affect, promotion focus, prevention focus, action readiness, optimism, and cooperation) are presented in **Table 3**. We found main effects of power on positive affect, *F*(1,68) = 6.26, *p* = 0.015; $\eta_{p}^{2}$ = 0.084, and promotion focus, *F*(1,68) = 6.39, *p* = 0.014; $\eta_{p}^{2}$ = 0.086. Replicating earlier findings (Anderson and Berdahl, 2002; Keltner et al., 2003), participants in the high power condition scored higher on positive affect (*M* = 4.47, SD = 0.71) and promotion focus (*M* = 4.95, SD = 0.86) than participants in the low power condition (*M* = 4.02, SD = 0.80; and *M* = 4.33, SD = 1.21 respectively).

**Table 3:** Self-report measures as a function of power and stability.

		Low Power		High Power	
		Unstable	Stable	Unstable	Stable
Positive affect	*M* SD	4.11 0.68	3.94 0.91	4.55 0.66	4.40 0.78
Negative affect	*M* SD	2.64 1.30	2.66 1.36	2.31 0.80	2.57 1.30
Promotion focus	*M* SD	4.56 1.27	4.11 1.14	5.19 0.86	4.71 0.82
Prevention focus	*M* SD	5.41 1.19	4.92 0.94	5.33 0.86	5.37 1.00
Action-readiness	*M* SD	4.29 0.75	4.36 0.93	4.50 0.91	4.59 0.90
Optimism	*M* *SD*	5.12 1.11	4.56 1.03	4.80 1.31	5.35 0.93
Cooperation	*M* SD	4.76 0.89	5.08 0.93	4.94 1.26	5.37 1.05

*Scales run from 1 (“not at all”) to 7 (“very strong”).*

**FIGURE 3:**
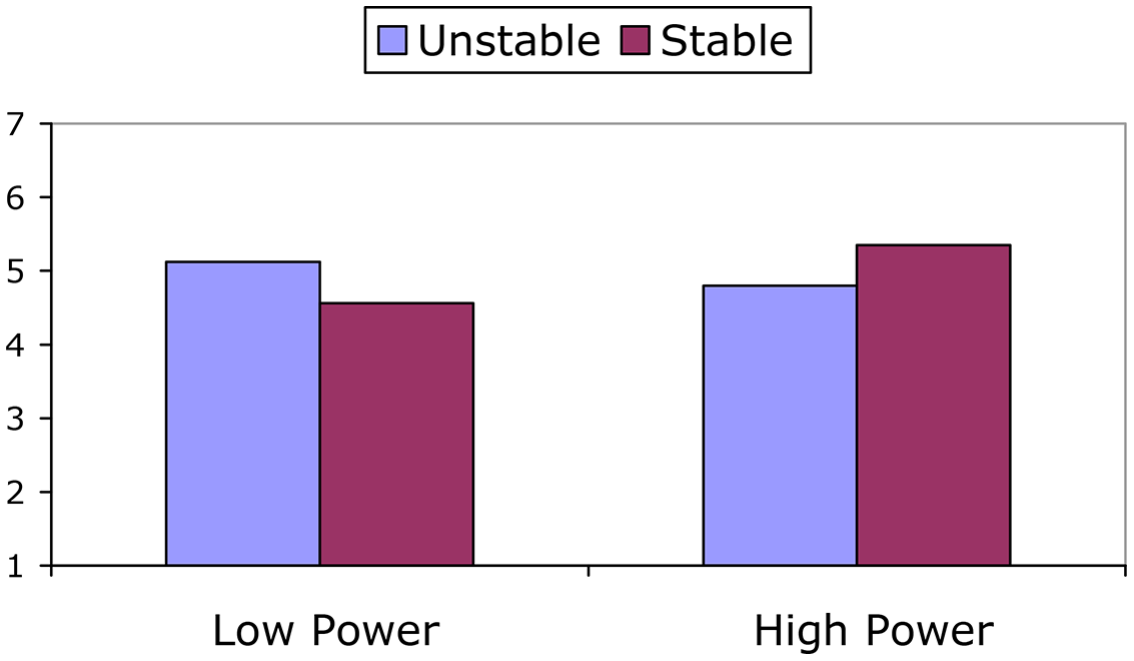
**Optimism as a function of power and power stability**.

For optimism there was a significant interaction between power and stability, *F*(1,68) = 4.63, *p* = 0.035; $\eta_{p}^{2}$ = 0.064. This interaction is displayed in **Figure 3**. As can be seen in the figure, when power relations were stable, participants in the low power condition were less optimistic than participants in the high power condition, *F*(1,68) = 4.83, *p* = 0.031; $\eta_{p}^{2}$ = 0.066; when power relations were unstable, however, these differences between those with high vs. low power did not emerge, *F*(1,68) = 0.75, *p* = 0.391; $\eta_{p}^{2}$ = 0.011.

There were no other significant effects on the self-report measures, *F*s < 2.28, *p*s > 0.135.

## Discussion

In the current research we examined the influence of the stability of power relations on CV-markers of motivation during a dyadic task. Three results are particularly noteworthy. First, participants in the low power condition were relatively more challenged when power differences were unstable than when they were stable, which was evident from higher CO and TCI in the former than in the latter case. Second, participants in the high power condition were relatively more threatened (as evident from high TPR) when power differences were unstable than when they were stable. Both these observations provide some evidence for our hypothesis. Finally, participants in the low power condition showed CV signs of *dis*engagement from the task when their position was stable. In the following we discuss the implications of these findings for work on power, health, and team performance.

The current research shows that having power is not always a positive state that is related to approach tendencies. That is, when power holders could possibly lose their privileged position they showed a maladaptive CV pattern, indicative of threat. In the context of motivated performance, the state of threat has been conceptualized as a conflict between approach and avoidance tendencies (Blascovich, 2008a). Thus, the current work adds a physiological dimension to work showing important moderators of the power–approach relationship (Maner et al., 2007; Lammers et al., 2008). This previous work has indicated that when power differences are perceived to be illegitimate or unstable, power holders tend to display a variety of avoidance related tendencies, like becoming more risk-aversive. Future work could examine whether the currently demonstrated physiological states function as mediators between power (in)stability and these behavioral consequences.

In a more general sense, the current work shows that having power does not necessarily lead to unconstrained freedom and pleasure, but that having power can actually be quite demanding. These demands of power can stem from uncertainty about one’s position, as illustrated in the current research, but also from other sources, like the meaning of power (i.e., how power is cognitively construed). Recent research shows that power holders can construe their power as an *opportunity* but also as a *responsibility* (Sassenberg et al., 2012). Power-holders who construe their power as an opportunity experience freedom and feel enabled to do what they want, while power-holders who construe their power as a responsibility experience the inner demand to do what is needed, and feel privileged and committed to act against impediments to the adherence to values and standards. There is evidence that power construed as responsibility is more demanding (and therefore often less attractive; Sassenberg et al., 2012) than power construed as responsibility. In effect, when exercising their power, power holders who were led to construe their power as a responsibility displayed a CV response profile indicative of threat, while power holders who construed their power as an opportunity displayed a CV response profile indicative of challenge (Scholl et al. under review). Thus, this work on the meaning of power fits nicely with the general conclusion of the current work, namely that it is not always “great to be the boss,” but that having power can sometimes be a burden.

The importance to move beyond main effects and take into account what power *means* is also in keeping with one of the main conclusions from an extensive review of work on the relationship between rank and health in primates (Sapolsky, 2005). As this work shows, the relationship between rank and stress is complex, and rank is in itself an imperfect predictor of health-related neuro-endocrine stress responses. Sapolsky (2005) discusses the stability of ranks among the chief moderators of the rank–health relationship. He concludes that when a hierarchy is stable the low-ranked primates display the strongest neuroendocrine signs of stress but that when the hierarchy is unstable the highly ranked primates display strongest signs of stress. Although the current work addressed a different population (humans) and a different physiological process (CV responses), drawing a parallel with the analysis of Sapolsky (2005) seems to some extent justified, also given the negative health consequences of the threat CV profile (see Blascovich, 2008b for a discussion). The identification of specific subgroups that are most vulnerable for stress during social interactions, e.g., in work settings, can serve as a basis for more specifically targeted interventions.

The current results do also have implications for interpersonal behavior in teams, and how these teams in turn perform. Apart from a CV threat profile, we also found that unstable power erased the self-reported tendency for optimism that is commonly found in those with high power and one of the hallmarks of charismatic leadership and the ability to engage in the pursuit of challenging goals. These findings resonate with prior research revealing that individuals who are insecure about their position in a work group, display dysfunctional task behavior, for instance by rejecting valid contributions made by others (Rink and Ellemers, 2015). In relation to this, the CV threat profile has also been related to suboptimal decision-making, as threatened persons become rigid (De Wit et al., 2012; see also Kassam et al., 2009; Jamieson et al., 2014). In sum, role insecurity might not only have long term health implications for those in power, but may also have immediate effects on task performance, and undermine broader social relations, with decreased productivity as a likely consequence.

So far we have mainly focused on the implications of the results for (unstable) high power. However, it is also important to look at the “other side of the coin,” namely the motivational processes in those with low power. The current results indicated that the prospects of change (i.e., instability) stimulate strong task engagement and benign CV arousal (challenge) in the powerless. However, when power was *stable* the powerless showed a tendency to disengage from the task as indicated by unchanged PEP. Thus, the current work shows that it is important to offer at least some prospects to those who have low power in team situations, in order to keep them positively involved in the task at hand (Ellemers et al., 2013).

At this point one may wonder about the extent to which the current results fit with the results of our earlier research where we found threat in the case of low power and challenge in the case of high power (Scheepers et al., 2012). That is, one might argue that the stable conditions in the current design are the ones that come closest to the low and high power conditions in our previous work where we did not manipulate power stability. While in our previous studies we found threat among those low in power, in the current study we found disengagement in the stable low power condition. Furthermore, while in our previous studies we found challenge among those high in power, in the current study the CV response profile for participants in the stable high power condition is more ambiguous (increased CO but stable TPR). We argue, however, that in both cases methodological differences can account for the asymmetry in results.

Regarding disengagement vs. threat in the (stable) low status condition it should be noted that in the current paradigm it was the low power person’s task to simply follow the orders that were given by the power-holder, which does not seem to be very engaging, especially in the absence of ways to improve one’s position. In contrast, in e.g., our previous negotiation study (Scheepers et al., 2012, Experiment 2), the low power negotiator might still have been engaged in the negotiation in order to reach the best deal possible, despite having relatively low resources and, as a consequence, showing a threat CV profile. Another factor contributing to disengagement in the current study might be that we provided explicit feedback about power stability while in our earlier research (some of) the participants in a low power position might have actually seen some prospects to improve their position, and thus remained engaged, despite still being threatened at the same time.

Regarding the absence of a strong challenge response in the (stable) high status condition it should be noted that we did not find evidence for challenge in the form of strongly decreased TPR in any of the conditions in the current design. Thus, it seems that the current paradigm has moved participants in the direction of threat, which might be explained by the demanding nature of the currently used task. For example, we purposely kept the instructions for the task somewhat vague (“design your ideal house”), to provide the power holder with some freedom to exercise his or her power. However, this might also have resulted in some task ambiguity. In addition, although participants had a few minutes to explore the “Sweet home 3D^®^” program, this might have been insufficient to get a good impression of all the options. Finally, some functions, like rotating the model, were a bit delayed, due to the limited memory capacity of the computer we ran the program on. Together, these task features and aspects of the procedure may have increased task uncertainty, introducing this as a source of threat across the board, which made it more difficult for a CV challenge response to emerge.

Finally, it should also be noted that despite that the patterns of results were in line with our hypotheses, some of the effects were just marginally significant. For example, even though the interaction on the TCI was significant and the pattern of means was in keeping with our hypotheses, the specific simple main effect tests failed to reach the conventional level of significance. This might be explained by the relatively low statistical power, in particular for the tests on the CV responses, which was due to signal loss during CV recording. Despite that the current effects are not extremely strong, we remain confident in their validity not in the last place because they nicely fit with the broader literature. That is, the effects are in keeping with work on neuroendocrine responses to unstable hierarchies (Sapolsky, 2005) and also our earlier research on CV responses to stable and unstable inter-group status hierarchies (Scheepers and Ellemers, 2005; Scheepers, 2009). However, we acknowledge that the current findings are preliminary and need to be replicated in future research.

The current work has illustrated that having power does not always lead to approach and benign physiological responses, but can be rather threatening when one’s powerful position can possibly change. Moreover, while low power can lead to disengagement when power is stable it can also lead to increased engagement and challenge when power is unstable. These results add (a physiological dimension) to the examination of moderators of the power-approach relationship, and can have implications to health and performance in work- and other team settings. Follow-up research should examine how CV responses as a function of (un)stable power differences mediates performance and other (behavioral) outcomes.

## Conflict of Interest Statement

The authors declare that the research was conducted in the absence of any commercial or financial relationships that could be construed as a potential conflict of interest.

## Footnotes

1 For eight persons all data was excluded from analyses due to technical errors (6) or suspicion (2). This means that we have data of 72 participants for the manipulation checks and self-report measures. The error terms of the ANOVAs that were conducted on these measures have 68 degrees of freedom. Regarding the baseline there was missing HR, PEP, and CO data for one participant. Regarding the speech task there was missing HR, PEP, and CO data for two persons. There was no overlap in missing data during the baseline and the speech task. Thus, a total of three participants had missing HR, PEP, and CO for either the baseline or speech task. As a consequence, the error terms of the ANCOVAs on task HR, PEP, and CO, in which baseline levels of the respective measure were added as covariates, had 72 - 3 - 5 = 64 degrees of freedom. As indicated in the main text, for the calculation of TPR, both CO and blood pressure data are necessary. Regarding the baseline there was missing TPR for seven participants, and regarding the speech task there was missing TPR for 16 participants; for six of these cases baseline TPR was also missing. As a consequence, the error term of the ANCOVA on task TPR, in which baseline TPR was added as a covariate, had 72 - 17 - 5 = 50 degrees of freedom.
